# Human lysyl-tRNA synthetase phosphorylation promotes HIV-1 proviral DNA transcription

**DOI:** 10.1093/nar/gkad941

**Published:** 2023-11-02

**Authors:** Yingke Tang, Ryan T Behrens, Corine St Gelais, Siqi Wu, Saravanan Vivekanandan, Ehud Razin, Pengfei Fang, Li Wu, Nathan Sherer, Karin Musier-Forsyth

**Affiliations:** Department of Chemistry and Biochemistry, Ohio State University, Columbus, OH, USA; Center for Retrovirus Research, Ohio State University, Columbus, OH, USA; Center for RNA Biology, Ohio State University, Columbus, OH, USA; McArdle Laboratory for Cancer Research, Institute for Molecular Virology, & Carbone Cancer Center, University of Wisconsin, Madison, WI, USA; Center for Retrovirus Research, Ohio State University, Columbus, OH, USA; Center for RNA Biology, Ohio State University, Columbus, OH, USA; Department of Veterinary Biosciences, Ohio State University, Columbus, OH, USA; State Key Laboratory of Chemical Biology, Shanghai Institute of Organic Chemistry, Chinese Academy of Sciences, China; Cellular and Molecular Mechanisms of Inflammation Program, National University of Singapore and The Hebrew University of Jerusalem (NUS–HUJ), Singapore; Department of Biochemistry and Molecular Biology, Institute for Medical Research Israel-Canada, The Hebrew University of Jerusalem, Israel; State Key Laboratory of Chemical Biology, Shanghai Institute of Organic Chemistry, Chinese Academy of Sciences, China; Department of Microbiology and Immunology, Carver College of Medicine, University of Iowa, Iowa City, IA, USA; McArdle Laboratory for Cancer Research, Institute for Molecular Virology, & Carbone Cancer Center, University of Wisconsin, Madison, WI, USA; Department of Chemistry and Biochemistry, Ohio State University, Columbus, OH, USA; Center for Retrovirus Research, Ohio State University, Columbus, OH, USA; Center for RNA Biology, Ohio State University, Columbus, OH, USA

## Abstract

Human lysyl-tRNA synthetase (LysRS) was previously shown to be re-localized from its normal cytoplasmic location in a multi-aminoacyl-tRNA synthetase complex (MSC) to the nucleus of HIV-1 infected cells. Nuclear localization depends on S207 phosphorylation but the nuclear function of pS207-LysRS in the HIV-1 lifecycle is unknown. Here, we show that HIV-1 replication was severely reduced in a S207A-LysRS knock-in cell line generated by CRISPR/Cas9; this effect was rescued by S207D-LysRS. LysRS phosphorylation up-regulated HIV-1 transcription, as did direct transfection of Ap4A, an upstream transcription factor 2 (USF2) activator that is synthesized by pS207-LysRS. Overexpressing an MSC-derived peptide known to stabilize LysRS MSC binding inhibited HIV-1 replication. Transcription of HIV-1 proviral DNA and other USF2 target genes was reduced in peptide-expressing cells. We propose that nuclear pS207-LysRS generates Ap4A, leading to activation of HIV-1 transcription. Our results suggest a new role for nuclear LysRS in facilitating HIV-1 replication and new avenues for antiviral therapy.

## Graphical Abstract

**Graphical Abstract:**
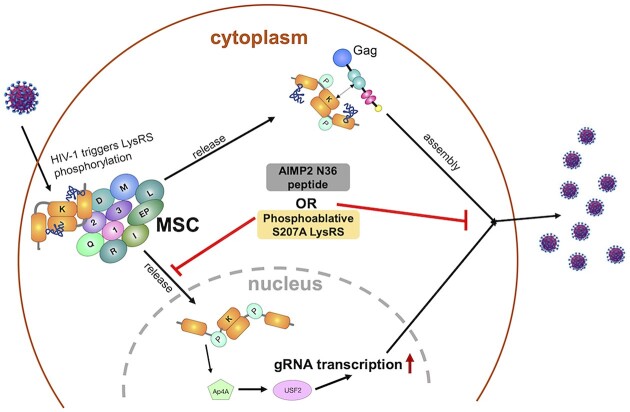

## Introduction

During human immunodeficiency virus type 1 (HIV-1) infection, the virus hijacks numerous host cell factors for replication. Host viral-dependency factors are potential therapeutic targets for anti-HIV-1 treatment (1–11).

Human lysyl-tRNA synthetase (LysRS) serves a key function in tRNA aminoacylation as part of the host's translational machinery; this aminoacyl-tRNA synthetase is also packaged into HIV-1 particles together with cognate tRNA^Lys^ (12). The human tRNA^Lys,3^ isoacceptor is annealed to the HIV-1 genomic RNA (gRNA) primer binding site (PBS) and serves as the reverse transcription primer (12–14). LysRS has been shown to interact with the HIV-1 Gag polyprotein directly to facilitate selective tRNA^Lys^ incorporation (15,16). Viral packaging of LysRS is dependent on phosphorylation at Ser207 (pS207-LysRS). A phosphoablative S207A-LysRS variant was no longer packaged and impaired progeny virion infectivity (17). Unexpectedly, pS207-dependent nuclear re-localization of LysRS during HIV-1 infection was also observed and MAPK/extracellular signal-regulated kinase (MEK) inhibitor U0126, blocked the nuclear localization of LysRS; however, the role of nuclear LysRS in HIV-1 replication in cells remains unknown.

In higher eukaryotes, LysRS is associated with a multi-aminoacyl-tRNA synthetase complex (MSC) consisting of nine aminoacyl-tRNA synthetases and three aminoacyl-tRNA synthetase complex-interacting multifunctional proteins (AIMPs). Within the MSC, LysRS and other tRNA synthetases function to charge amino acids to their cognate tRNAs. Upon various cellular stimuli, LysRS and other MSC components are released from the MSC to carry out non-canonical functions beyond translation (18–20); phosphorylation is often a key determinant of this functional dualism.

LysRS was previously shown to be phosphorylated on Ser207 upon IgE stimulation of mast cells (21). This resulted in dissociation from the MSC and trafficking to the nucleus where microphthalmia transcription factor (MITF) or upstream transcription factor 2 (USF2) were subsequently activated (21,22). LysRS Ser207 phosphorylation triggers a closed-to-open conformational change that reduces tRNA aminoacylation activity but stimulates a second catalytic function - synthesis of the dinucleotide signaling molecule, diadenosine tetraphosphate (Ap4A) (23). Ap4A binds to the histidine triad nucleotide-binding protein 1 (HINT1), resulting in release of transcriptional factors MITF or USF2 and transcriptional activation of target genes (24). We hypothesize that the nuclear re-localization of LysRS during HIV-1 infection may lead to regulation of host and viral gene transcription.

HIV-1 transcription is driven by the viral promoter in the 5′ long terminal repeat (5′ LTR) and its cognate transcription factors. The *cis*-acting elements in the 5′ LTR allow transcription factor binding, which controls both HIV-1 replication and latency. USF1/USF2 are among the transcription factors shown to regulate 5′-LTR activity (25,26). These factors are recruited to the 5′ LTR in response to Ras-MAPK signaling in CD4+ T cells, but the mechanism by which this occurs is still unknown (25). Phosphorylation of LysRS is also MAPK-dependent and pS207-LysRS has been reported to bind USF2 in the nucleus (27). Herein, we examine the hypothesis that the nuclear pool of pS207-LysRS regulates HIV-1 transcription via activation of USF2.

To probe the nuclear function of pS207-LysRS in HIV-1 replication, we investigated the effect of homozygous S207A-LysRS knock-in in CD4+ Jurkat T cells on HIV-1 transcription and long-term replication. Our results demonstrate a critical nuclear function of LysRS in the HIV-1 lifecycle and suggest a new potential anti-HIV strategy.

## Materials and methods

### Materials

Details of antibodies, PCR primers, plasmids, cell lines, other reagents and software are found in Supplementary Tables S1–S4.

### Cell culture and media

HEK293T cells were grown in Dulbecco's modified Eagle's medium (DMEM) supplemented with 10% (vol/vol) fetal bovine serum (FBS), 100 IU/ml penicillin, and 100 μg/ml streptomycin (complete DMEM). Jurkat E6-1, Jurkat^S207A^ E6-1 and SupT1 cells were maintained in Roswell Park Memorial Institute (RPMI) medium supplemented with 10% (vol/vol) fetal bovine serum (FBS), 100 IU/ml penicillin and 100 μg/ml streptomycin (complete RPMI). Jurkat^S207A^ E6-1 TripZ-LysRS^WT^, Jurkat^S207A^ E6-1 TripZ-LysRS^S207A^, Jurkat^S207A^ E6-1 TripZ-LysRS^S207D^ were maintained in complete RPMI with 0.25 μg/ml puromycin. THP1 cells were cultured in complete RPMI with sodium pyruvate (X100, 100 mM). GHOST X4/R5 cells were maintained in complete DMEM, 1 μg/ml puromycin, 500 μg/ml Geneticin, and 100 μg/ml hygromycin B.

### Genome engineering of Jurkat E6-1 cells

Alt-R™ recombinant S.p. Cas9 nuclease-3NLS (IDT, #1074181), Alt-R™ CRISPR/Cas9 crRNAs (IDT), ATTO™-550 labeled Alt-R™ tracrRNA (IDT, #1075928), and Alt-R™ Cas9 electroporation enhancer reagents were prepared according to the manufacturer's instructions. tracrRNA-crRNA and Cas9-containing complexes were assembled according to the manufacturer's instructions. The 119-nt single-stranded oligodeoxynucleotide (Sigma) donor template for homology-directed repair (HDR) was reconstituted in TE buffer. HDR donor template for LysRS.S207A: 5′-CCA AGA AGG GTG AGC TGA GCA TCA TTC CGT ATG AGA TCA CAC TGC TG**g Cg**C CCT Gcc TtC AcA TGc TgC CaC ATC TTC ACT TTG GCC TCA AAG ACA AGG TAA GCG TTC TTG GCC TCC TA-3′. Cas9-tracrRNA-crRNA complexes and HDR templates were delivered to Jurkat E6-1 cells using the Neon® Transfection System and Neon® Transfection 10 μL Kit (Invitrogen) according to manufacturer's instructions. Electroporation parameters were 1600 V, 10-ms pulse width, 3 pulses and cells were cultured post-electroporation in antibiotic-free media (RPMI 1640 supplemented with 10% FBS). Single cell clones were isolated by fluorescence-associated cell sorting (FACS). PCR products were amplified from genomic DNA using GoTaq Flexi PCR kits (Promega) and the following screening primers: Forward screening primer: 5′-GCA ACA CAG CAA TAG GCT G-3′. Reverse screening primer: 5′-ATC ACG TCA GGC AAG GAA CT-3′. Restriction enzyme digests and Sanger sequencing of PCR products were used to screen Jurkat cells for knock-in genome edits.

### Cell lysis and western blot analyses

Cell pellets were lysed in cell lysis buffer (CLB) (Cell Signaling Technology), or RIPA lysis buffer (150 mM NaCl, 1% Nonidet *P*-40, 0.5% sodium deoxycholate, 0.1% SDS and 25 mM Tris pH 7.4), both supplemented with a protease inhibitor cocktail (Sigma-Aldrich). For phosphorylated protein analysis, phosphatase inhibitor cocktail (Cell Signaling Technology, product #58715) was added to the lysis buffer. Cells were lysed for 30 min on ice, followed by centrifugation for 30 min at 13 200 rpm at 4 °C. The supernatants were collected and mixed with Laemmli sample buffer (4×) or 4× SDS Sample Loading Buffer (Sigma-Aldrich). Following SDS-PAGE, proteins were transferred onto polyvinylidene difluoride (PVDF) membranes (Bio-Rad). Primary antibodies were used at 1:1000 dilution in 5% bovine serum albumin in TBS-tween (BSA/TBST). Secondary antibodies were diluted at 1:10000. Blotting signal was detected by using Super-Signal chemiluminescence substrates (Thermo Fisher, cat#34579). Images were acquired by GE Amersham Imager 600. A complete list of antibodies used in this work and their sources is shown in Supplementary Table S1.

### RNA extraction and quantitative reverse-transcription PCR

Total cellular RNA was extracted by using TRIZol reagent (Invitrogen, Cat# 15596018) according to the manufacturer's instruction, followed by DNA digestion using TURBO DNase (Invitrogen, Cat# AM2239). Reverse transcription (SuperScript IV, Invitrogen, Cat# 18090050) was primed by random hexamer and qPCR was performed using PowerUp SYBR green master mix (Thermo Fisher Cat#A25741) with specific primers shown in Supplementary Table S2. Transcript levels were normalized by 18S rRNA or β-actin cDNA.

### Dual luciferase assays

The HIV-1 Firefly-Luciferase vector (pGL3-LTR-luc) and the TK promoter-driven Renilla-Luciferase reporter plasmid (pRenilla-TK) were previously described (28). Cells were harvested and pelleted at 48 h post transfection. Dual-luciferase assays were conducted using the dual luciferase reporter system kit (Promega Cat #E1910) according to the manufacturer's instruction. HIV-1 LTR activity was calculated using the firefly luciferase readout normalized by the renilla luciferase readout.

### Cell treatment and transfection

Transfection of HEK293T cells was performed using the CaCl_2_ and BES-buffered saline method as described previously (29) or using polyethylenimine (PEI). DNA plasmid and Ap4A transfection in Jurkat cells were carried out using Lipofectamine™ 3000 transfection reagent (Thermo Fisher Cat #L3000001) according to the manufacturer's protocol.

### pTripZ-LysRS plasmid cloning and stable cell line generation

To generate stable Jurkat^S207A^ cell lines with exogenous LysRS expression, DNA encoding C-terminally Flag-tagged human LysRS (WT, S207A, S207D) was obtained from codon optimized pCDNA3.FLLysRS (WT, S207A, S207D) plasmids using PCR (17). (primers: Fwd:5′AAC CGT CAG ATC GCA CCGG ATG GCT GCT GTG CAA GCC GCA-3′; Rev: 5′ CCG GCG CGG AGG CCA GGT TCT TTC CGC CTC AGA -3′). C-Flag-LysRS constructs were cloned into a doxycycline-inducible Lentiviral vector pTripZ (Dharmacon). The pTripZ plasmid was doubly-digested with restriction enzymes AgeI and MluI, followed by assembly with C-Flag-LysRS using NEBuilder® HiFi DNA assembly kit (New England BioLabs). Sequences of pTripZ-LysRS plasmids were confirmed by Sanger sequencing. pTripZ-LysRS, packaging plasmid pDeltaR8.2 and envelope plasmid pMD2.G were co-transfected into HEK293T Lenti-X cells by lentivirus transduction according to the manufacturer's protocol to generate Jurkat^S207A^ stable cell lines with exogenous LysRS expression. Stable cell lines were grown in complete RPMI 1640 medium with 0.25 μg/ml puromycin.

### HIV-1 production and cell infection

Single-cycle HIV-1 luc and HIV-1 GFP stocks were generated by co-transfection HEK293T cells (5 × 10^6^) of 10 μg of proviral DNA pNL4-3 LucR + E- or pNL4-3ΔEnv EGFP, along with 2 μg of pMD2.G expressing vesicular stomatitis glycoprotein (VSV-G). Replication competent HIV-1 stocks were generated by using 10 μg proviral DNA construct pNL4-3. HEK293T Lenti-X cells were seeded and transfected by PEI in 10-cm culture dishes. At 72 h post-transfection, cell-free supernatant was harvested and filtered through 0.45 μm syringe filters and stored in –80 °C. Virus titers were determined by GHOST X4/R5 cell titration as previously described (17). Cells were infected with freshly thawed HIV-1-containing supernatants and incubated for 6–24 h prior to exchanging the medium.

### HIV-1 replication and p24 ELISA assays

Jurkat cells (2 × 10^6^) were cultured in 5 ml of complete RPMI 1640. Cells were infected with HIV-1_NL4-3_ virus stock at MOI 0.2 and the medium was exchanged 12 h post infection. Virus-containing supernatants were collected and stored in -20 °C every 2 days beginning day 2 post infection. To maintain the long-term culture, cells were passaged and split at a 1:5 ratio every 4 days. To quantify the released HIV-1 particles, 450 μl of cell-free supernatant was used in p24 ELISA assays (ZeptoMetrix ELISA kit) according to the manufacturer's instructions.

### Nuclear-cytoplasmic fractionation

The protocol for nuclear fractionation was modified from a previous method (30). Briefly, cells were washed with ice-cold PBS, pelleted in 1.5 ml eppendorf tubes and lysed in 360 μl 0.1% NP-40 in PBS. Total lysate (90 μl) was mixed with 30 μl 4× Leammli sample buffer (200 mM Tris pH 6.8, 8% SDS, 40% glycerol, 20% $\beta$-mercaptoethanol, 0.2% bromophenol blue). The remaining lysate was centrifuged at 13 200 rpm for 1 min to precipitate the insoluble nuclear fraction. A portion of the supernatant (90 μl) was saved as the cytoplasmic fraction. Insoluble nuclear pellets were washed 3 times with 500 μl 0.1% NP-40 in PBS and lysed in 100 μl 1× Leammli sample buffer. Total and nuclear lysate fractions were sonicated for 15 sec with 20% energy strength (Ultrasonic Processors 130-WATT) to break nuclei and release the nuclear protein.

### Co-immunoprecipitation Assays

Cell cultures for co-IP experiments were generally grown in 6-well plates. Approximately 5 × 10^6^ cells were harvested and lysed in 400 μl radioimmunoprecipitation assay (RIPA) lysis buffer or 1% TritonX-100, with addition of protease inhibitor cocktail (100×, Sigma-Aldrich, Cat# P8340) for 40 min on ice. In the co-IP assays of LysRS and USF2, cell lysates were sonicated for 15 sec with 20% energy strength (Ultrasonic Processors 130-WATT) to break nuclei and release nuclear proteins completely. Primary antibody (2.5 μl) and agarose beads (Santa Cruz Biotechnology, Protein A/G PLUS-Agarose, sc-2003) were incubated with cell lysates in 500 μl of 1% TritonX-100, with rotation overnight. The agarose beads were washed with 1 ml lysis buffer 3 times, pelleted at 3000 rpm, and boiled at 95 °C in 100 μl 1× Leammli sample buffer.

### MSCV-AIMP2-N36 cell line generation

Lentivirus containing the AIMP2-N36 gene was generated from HEK293TLentiX cells by using PEI transfection. Lentivector plasmids (5 μg) pMSCV-AIMP2-N36 or the control plasmid pMSCV-EGFP were co-transfected with 5 μg of pMoMLV-Gag and 2 μg of pMD2.G in a 10-cm culture dish. HEK293T and SupT1 cells were transduced by infection and selected in medium with 1 μg/ml puromycin.

### Quantification of tRNA^Lys3^, HIV-1 gRNA and 7SL RNA in HIV-1 virions by RT-qPCR

Virions were isolated from the supernatant by filtering through a 0.45 μm syringe filter followed by ultracentrifugation through a 25% sucrose cushion. Total viral RNA was extracted using TRIZol reagent (Invitrogen) according to the manufacturer′s instructions. DNA was digested using TURBO DNase (Invitrogen) at 37 °C for 30 min followed by inactivation of the enzyme at 85 °C for 10 min. Reverse transcription (SuperScript IV, Invitrogen) of cDNA was primed by random hexamers, followed by qPCR performed using PowerUp SYBR green master mix (Thermo Fisher) with specific primers (Supplementary Table S2). Transcript levels of tRNA^Lys3^ and HIV-1 gRNA were quantified using the 2^(-ΔΔCt) method normalized by 7SL RNA as described (31).

### Statistical analysis

Statistical analysis was performed using GraphPad Prism version 7. *P* values (two-tailed) were calculated using an unpaired *t* test as annotated in the figure legends.

## Results

### Endogenous LysRS phosphorylation at Ser207 is elevated during HIV-1 infection

**Figure 1.**
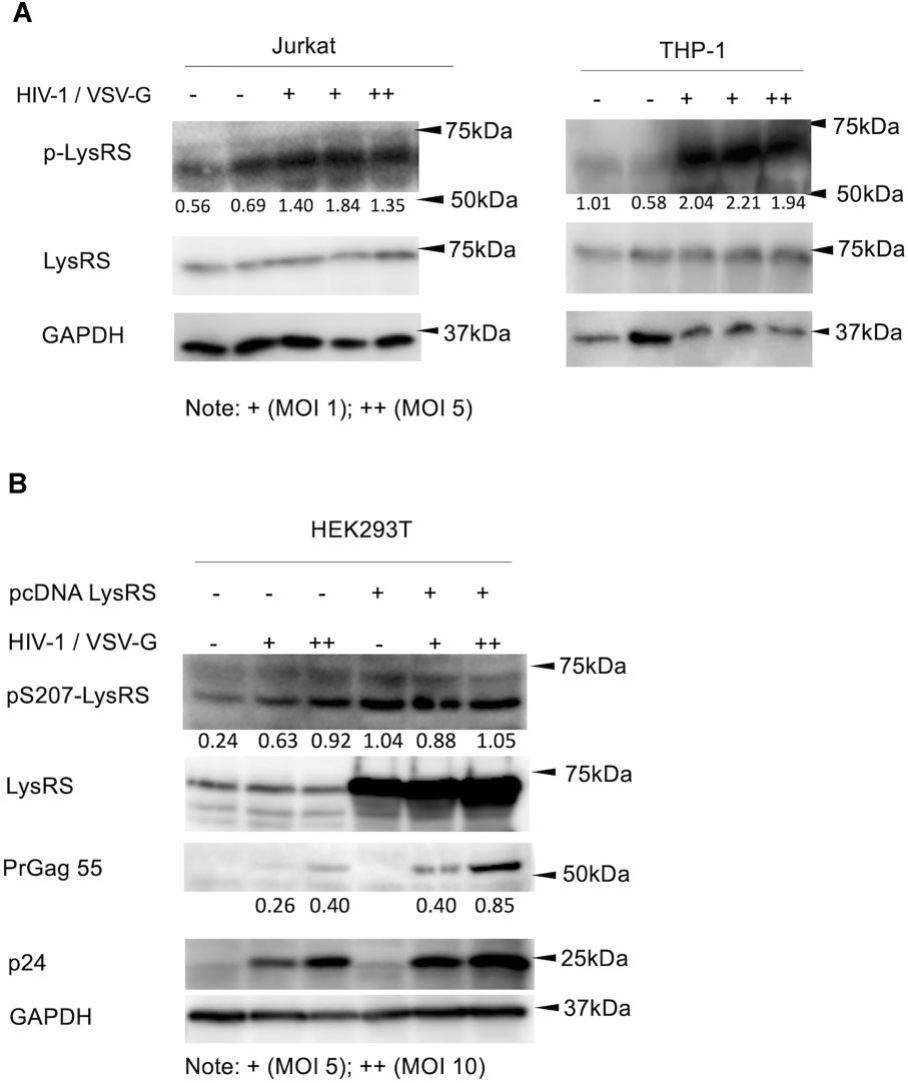
LysRS is phosphorylated at S207 and facilitates HIV-1 expression during HIV-1 infection. (**A**) Immunoblots probing phosphorylation on S207 of LysRS in Jurkat and THP-1 cells during HIV-1 infection. Endogenous LysRS was probed using anti-pS207 antibody (top) and anti-LysRS antibody (middle), in the absence (–) and presence of single-cycle HIV-1 infection at MOI 1 (+) and MOI 5 (++). Bands were quantified and intensity was normalized to the GAPDH loading control. (**B**) Immunoblot showing phosphorylation level of LysRS and HIV-1 protein expression in HEK293T cells during HIV-1 infection. In the absence (-) or presence (+) of overexpressed LysRS, cells were infected with single-cycle HIV-1 at MOI 5 (+) or MOI 10 (++). Pr55Gag represented the newly synthesized HIV-1 protein in infected cells, and p24 (HIV-1 CA) was from both incoming virions and mature virions in cells. The pS207-LysRS and Pr55Gag levels were quantified and normalized by loading control GAPDH.

To directly measure endogenous levels of LysRS S207 phosphorylation during HIV-1 infection, we used a pS207-LysRS-specific polyclonal antibody (32). Experiments were carried out in CD4 + Jurkat T cells and a monocytic THP-1 cell line (33). Cells were infected by single-cycle HIV-1 at increasing multiplicity of infection (MOI), harvested at 48 h post infection and analyzed by Western blotting (Figure 1A). The results indicate an increase in endogenous pS207-LysRS in both cases; the results are most clear in THP-1 cells where the levels of pLysRS in the absence of infection are very low. The major species migrates faster than the expected 68 KDa full-length LysRS band; both full-length and truncated LysRS species are phosphorylated (see also Supplementary Figure S1D). Truncated LysRS species have previously been shown to be packaged into HIV-1 (16). Overall, these results indicate an increase in the endogenous levels of pS207-LysRS upon HIV-1 infection.

HIV-1 Gag is a polyprotein critical for viral assembly at the plasma membrane of host cells. We found that overexpression of LysRS enhanced HIV-1 Gag production in single-cycle infection. HEK293T cells were transduced with single-cycle HIV-1 at an increasing MOI and harvested at 48 h post infection. The total cell lysates were analyzed by Western blotting. In the absence of exogenous LysRS expression, phosphorylation of endogenous LysRS at S207 was induced upon HIV-1 infection, as expected (Figure 1B, left three lanes). When we overexpressed LysRS in these cells, the overall LysRS was elevated, as expected, and the phosphorylation level was elevated regardless of the presence of HIV-1 infection (Figure 1B, right three lanes). The production of HIV-1 Gag protein (Pr55Gag) was also promoted by LysRS overexpression, suggesting a regulatory role of LysRS in HIV-1 replication beyond its reported function in primer packaging (17,34) and reverse transcriptase (RT) maturation (35).

### HIV-1 replication is suppressed in Jurkat S207A-LysRS knock-in cells

To investigate the role of LysRS S207 phosphorylation in the HIV-1 lifecycle without interfering with its essential function in translation, we generated a CRISPR/Cas9 knock-in cell line that only expresses S207A-LysRS. The phosphoablative S207A-LysRS mutant has previously been shown to maintain WT levels of tRNA aminoacylation capability *in vitro* (17); thus, we expected the knock-in (KI) cell line to be viable. Jurkat cells are a well-established model T cell line with known methods for CRISPR/Cas9 KI (36). Thus, we chose Jurkat cells for this work. To achieve site-directed KI, the DNA encoding LysRS was cleaved by the Cas9 protein and then repaired by homology-directed repair (HDR) with a DNA donor segment containing the mutated DNA sequence, as well as a KsaI restriction site to facilitate clone analysis (Supplementary Figure S1A). The PCR products from homozygous KI cell lines were cleaved by KasI, while the heterozygous clones resulted in partial cleavage (Supplementary Figure S1B). One of the homozygous clones, 4.10D, was selected for the studies described herein. As expected, Jurkat^S207A^ KI cells proliferated as well as parental Jurkat^WT^ cells (Supplementary Figure S1C). Using the anti-pS207-LysRS antibody, we confirmed the absence of increasing LysRS phosphorylation over the background signal observed in the Jurkat^S207A^ KI cells during HIV-1 infection (Supplementary Figure S1D).

**Figure 2.**
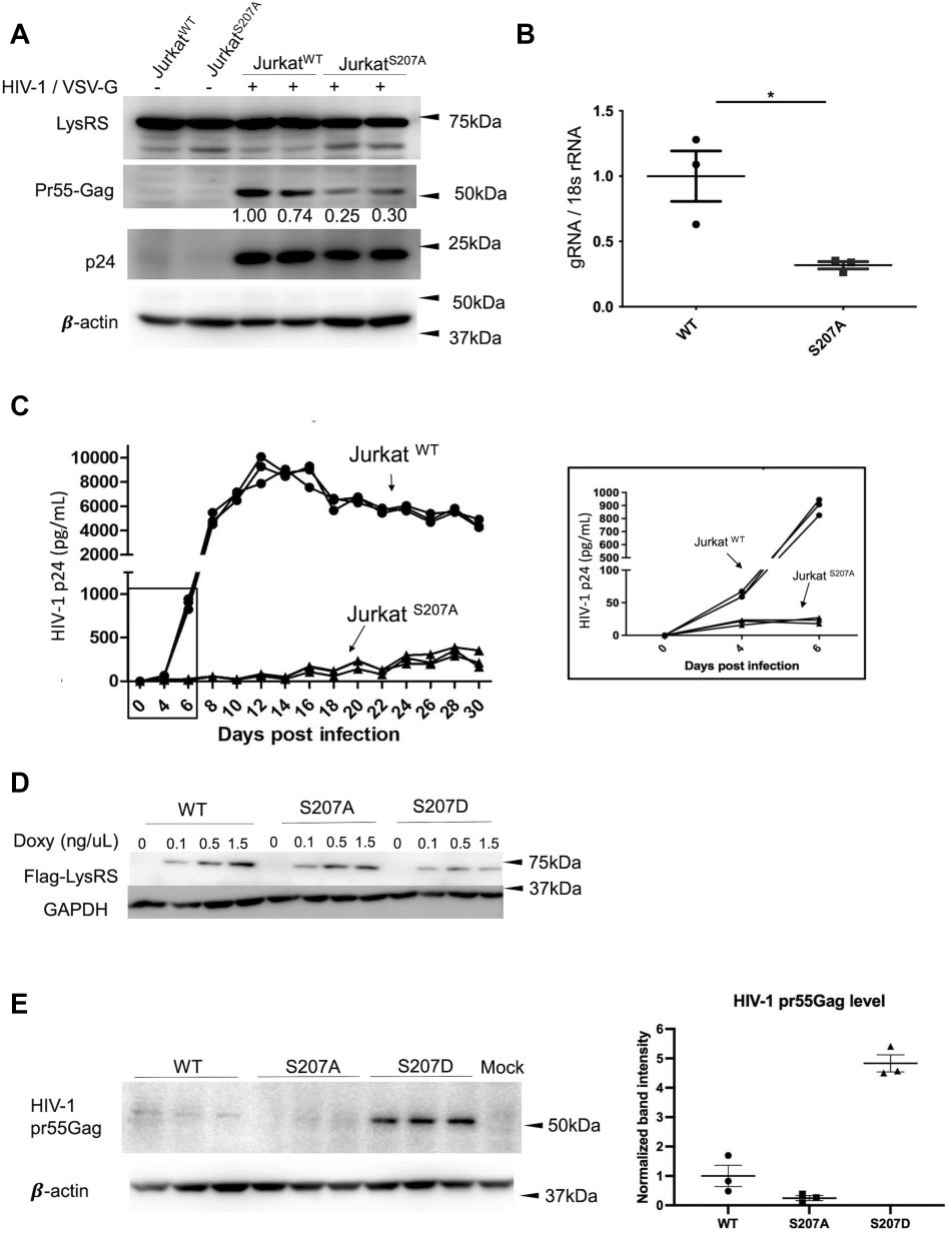
HIV-1 replication was significantly impaired in S207A knock-in Jurkat cells. (**A**) Immunoblots showing HIV-1 protein expression levels in Jurkat^S207A^ and Jurkat^WT^ cells during single-cycle HIV-1 infection (MOI 1). The pr55Gag protein levels were quantified by band intensity and normalized to β-actin. (**B**) RT-qPCR analysis of HIV-1 gRNA transcript levels in Jurkat^S207A^ and Jurkat^WT^ cells with single-cycle HIV-1 infection at MOI 1 (*P < 0.05). (**C**) HIV-1 replication curves in Jurkat^S207A^ and Jurkat^WT^ cells. Cells were infected by HIV-1_NL4-3_ at MOI 0.02 and split every 4 days. The replication of HIV-1_NL4-3_ was monitored by measuring the released HIV-1 p24 in cell culture supernatants by ELISA. The rectangle indicates the region shown in the expanded view on the right. (**D**) Immunoblots showing the expression of exogenous WT-, S207D- or S207A-LysRS in Jurkat^S207A^ cells before and after doxycycline induction (0.1, 0.5, 1.5 ng/μl). (**E**) Immunoblots showing the rescue of HIV-1 replication by WT-, S207D- or S207A-LysRS in Jurkat^S207A^ cells. The levels of Gag production during HIV-1_NL4-3_ infection (n = 3) in Jurkat^S207A^ -WT, Jurkat^S207A^ -S207A and Jurkat^S207A^ - S207D cells with 1.5 ng/μl doxycycline induction. The lane labeled Mock is Jurkat cells without HIV-1. The levels of Pr55Gag production were quantified and normalized by the loading control β-actin, as shown in the scatter plot on the right.

Experiments wherein Jurkat^S207A^ and Jurkat^WT^ were infected with single-cycle HIV-1, revealed that the production of newly synthesized HIV-1 Gag was suppressed in the KI cells compared with WT (Figure 2A and Supplementary Figure S1F). Endogenous LysRS expression and HIV-1 p24 capsid (CA) protein levels (in-coming) remain constant, as expected. HIV-1 transcription was similarly suppressed in the Jurkat^S207A^ cells to ∼25% of the Jurkat^WT^ levels (Figure 2B). Single-cycle infection performed with another pseudotyped virus, HIV-1 $\Delta$Env-EGFP/VSVG, which expresses an EGFP reporter, confirmed the reduction of HIV-1 infection in the S207A KI cells (Supplementary Figure S1E).

To investigate the long-term effect of S207A-LysRS KI in HIV-1 replication, we conducted a 30-day spreading infection assay. Jurkat^S207A^ and Jurkat^WT^ were infected with replication-competent HIV-1_NL4-3_ at MOI 0.02 and produced progeny virions, as detected by p24-based ELISA, starting at day 3 post infection. In Jurkat^WT^ cells, viral spread progressed through a burst stage to reach a peak (day 12), and then gradually decreased, as expected (days 14–30) (Figure 2C). In contrast, in the Jurkat^S207A^ cell cultures, even though the initial infection was successful and progeny virions were generated, neither a burst stage nor a peak was observed (Figure 2C). The released HIV-1 p24 levels only demonstrated an ∼2-fold increase by day 8 post infection demonstrating that viral spread was severely impaired in the Jurkat^S207A^ KI cells compared to WT cells.

To exclude off-target effects from CRISPR/Cas9 editing in Jukrat^S207A^ cells, we performed a rescue assay by introducing exogenous C-terminally flag-tagged WT-, S207A- and S207D-LysRS into the Jurkat^S207A^ knock-in cells using a lentiviral-transducing vector pTripZ. Transduced cells were selected by puromycin and the expression of exogenous WT or mutant LysRS was stably induced with doxycycline (Figure 2D). Following doxycycline induction (48 h), Jurkat^S207A^-WT, Jurkat^S207A^-S207A, and Jurkat^S207A^-S207D cell lines were infected with HIV-1_NL4-3_ and Gag levels were examined in cell lysate by Western blotting performed 5-days post infection. HIV-1 replication was rescued in Jurkat^S207A^-S207D cells, moderately rescued in Jurkat^S207A^-WT cells, but not rescued in Jurkat^S207A^ cells expressing S207A-LysRS (Figure 2E). These results confirmed the inhibitory effect of S207A-LysRS KI on HIV-1 infection and suggest a critical role for pS207-LysRS in HIV-1 replication.

### AIMP2-N36 peptide inhibits HIV-1 replication by binding LysRS

**Figure 3.**
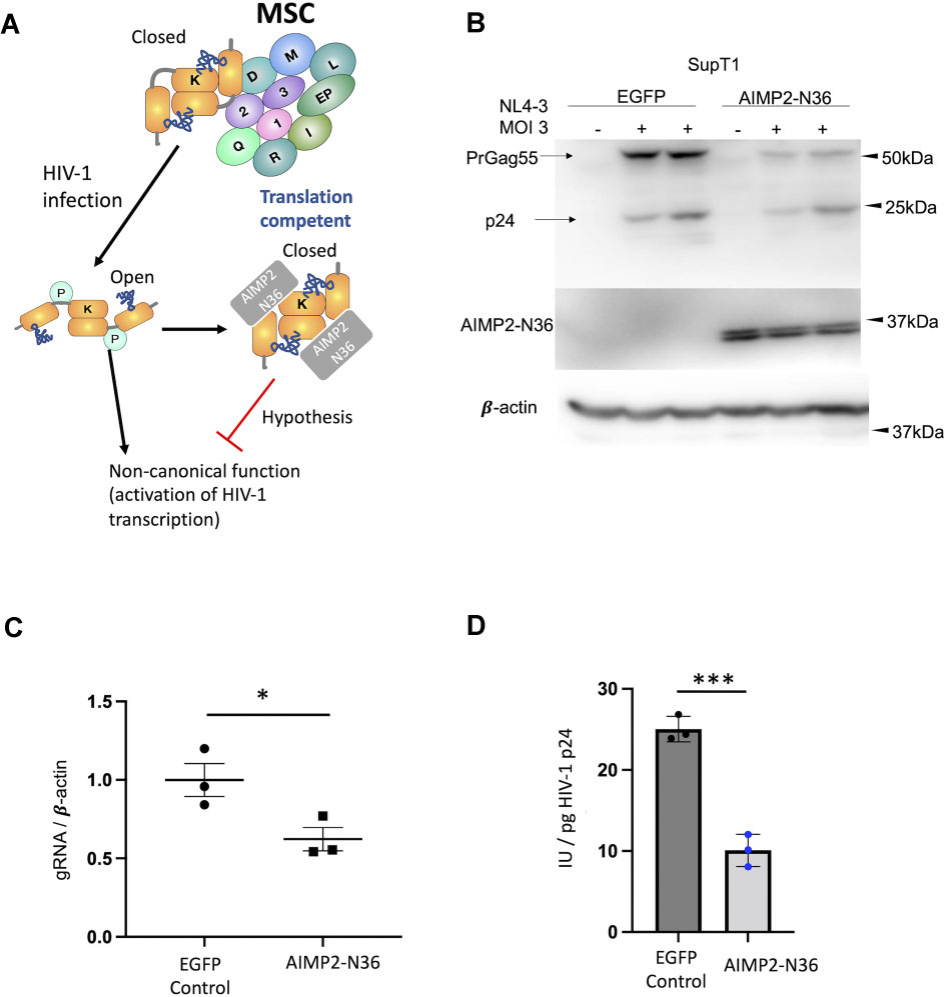
AIMP2-N36 peptide blocks pLysRS noncanonical function and suppresses HIV-1 replication. (**A**) Scheme showing the hypothesis being tested in this work. Translation-competent LysRS associates with the AIMP2 scaffold protein in the MSC. HIV-1 infection triggers phosphorylation and release of LysRS from the MSC and induces the closed-to-open conformational change. The open LysRS is incapable of charging tRNA but gains non-canonical functions including activation of HIV-1 replication. AIMP2-N36 peptide binds to released LysRS and locks it in the closed translational competent conformation, thereby suppressing HIV-1 replication. (**B**) Immunoblots showing the replication of HIV-1 in AIMP2-N36-expressing SupT1 cells and the SupT1-EGFP control cells. Cells were infected by HIV-1_NL4-3_ at MOI 3 and harvested at 4 days post infection. (**C**) RT-qPCR analysis of HIV-1 gRNA transcription levels in AIMP2-N36-expressing SupT1 cells and the SupT1-EGFP cells during single-cycle HIV-1 infection (*P < 0.05). (**D**) Graph showing the infectivity of progeny HIV-1 generated from in SupT1- AIMP2-N36 cells and the SupT1-EGFP control cells, presented in infectious units normalized by HIV-1 p24 (IU/pg HIV-1 p24) (***P < 0.0005).

In the MSC, LysRS dimers are bound by the scaffold protein AIMP2. The structure of the LysRS-AIMP2 subcomplex shows that the AIMP2 N-terminus (N36) is the site of LysRS binding (23,37); this interaction presumably locks LysRS in a closed conformation necessary for tRNA aminoacylation (38). Indeed, previous studies confirmed interaction of LysRS with an AIMP2-N36 peptide expressed in HEK293T cells (37). Upon S207 phosphorylation, LysRS dissociates from AIMP2 and is released from the MSC, undergoing a conformational change to form an open conformer that is aminoacylation defective (23). We hypothesized that overexpression of AIMP2-N36 may serve to block the function of LysRS in facilitating HIV-1 replication (Figure 3A).

To test this hypothesis, we expressed the AIMP2-N36 peptide fused with EGFP and an N-terminal Flag-tag in cells using a lentivector transducing system; an EGFP-only construct served as the control. The cell lines that stably expressed AIMP2-N36 or EGFP were selected in culture medium containing 1 μg/ml puromycin. We chose SupT1 cells for generating these stable cell lines, since SupT1 cells are characterized by high CD4+ expression on the cell surface; this feature increases the efficiency of HIV-1 infection.

The effect of the AIMP2-N36 peptide on HIV-1 replication was tested. AIMP2-N36- and EGFP-expressing SupT1 cells were infected with HIV-1_NL4-3_ and harvested at 4 days post infection. HIV-1 replication was suppressed in cells expressing the AIMP2-N36 peptide, compared with the EGFP control cells, as indicated by reduced Gag expression (Figure 3B). Genomic RNA was quantified by RT-qPCR in a single-cycle HIV-1 infection assay. The production of HIV-1 gRNA at 48 h post infection was suppressed in AIMP2-N36-expressing cells compared to the control (Figure 3C), suggesting that the peptide inhibited the nuclear function of LysRS in regulating HIV-1 transcription.

To test the effect of the AIMP2-N36 peptide on progeny virion infectivity, SupT1 cells expressing the peptide or EGFP control were spun down and the supernatants containing progeny virions were titrated using GHOST-X4/R5 cells, a reporter cell line that contains a HIV-1 LTR-driven GFP gene (39). GFP expression was analyzed by flow-cytometry and the results are plotted in Figure 3D. These results show that progeny virions are significantly (∼2-fold) less infectious when collected from cells overexpressing the AIMP2-N36 peptide.

### LysRS regulates HIV-1 transcription and the activity of USF2

**Figure 4.**
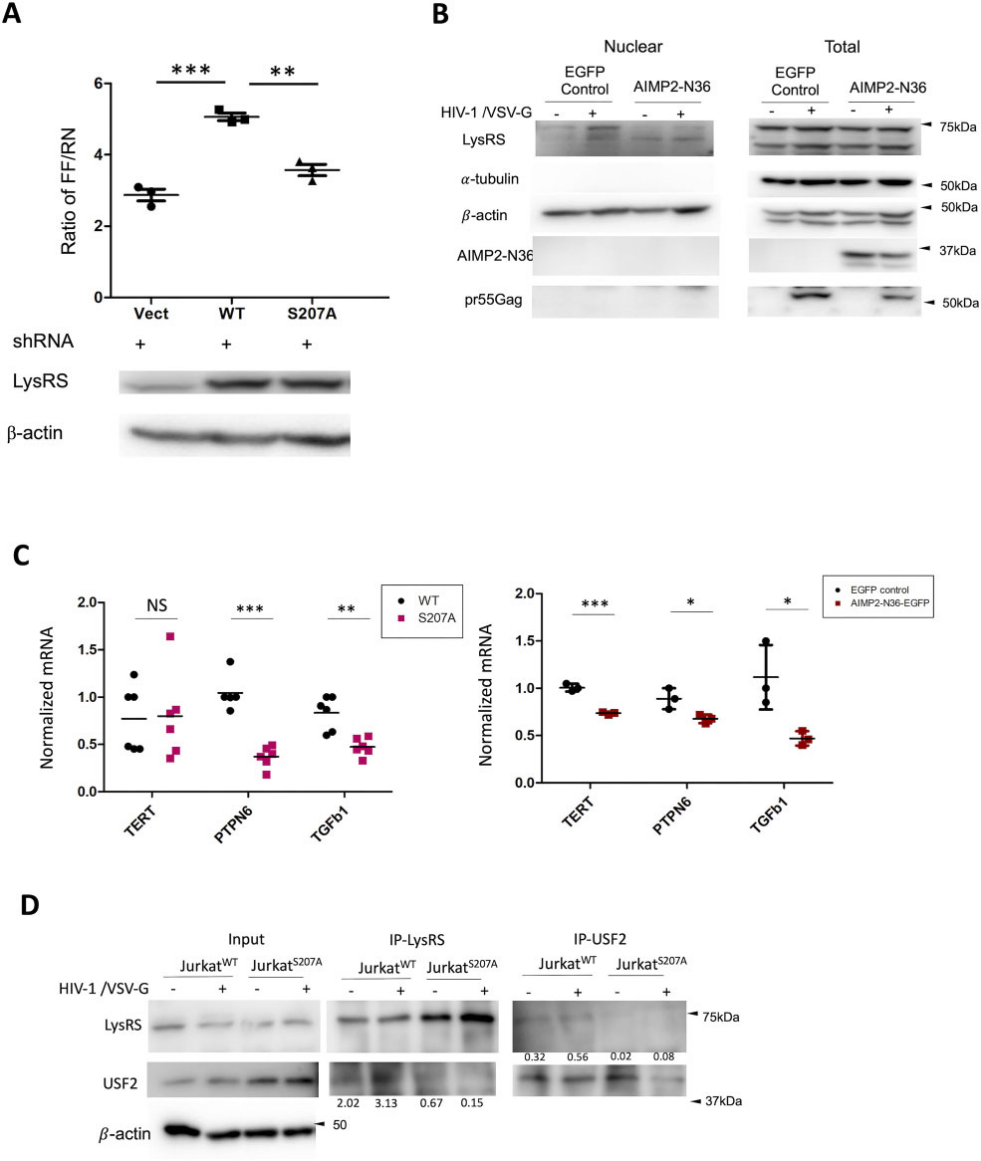
pLysRS relocates to nucleus and regulates HIV-1 transcription via USF2 pathway. (**A**) Normalized HIV-1 5′ LTR activity with overexpression of WT-LysRS, S207A-LysRS, or a vector control (vect) in HEK293T cells wherein endogenous LysRS is knocked down. The activity of the HIV-1 5′LTR promoter was examined using the dual-luciferase system described in Materials and methods. (**B**) Nuclear fractionation assay of HEK293T cells stably expressing AIMP2-N36-EGFP peptide or an EGFP control at 48 h post single-cycle HIV-1 infection at MOI 1. The nuclear and total cellular lysates were analyzed by western blotting. a-tubulin served as the cytosolic marker and β-actin is expected to be present in both the nucleus and cytoplasm. (**C**) Scatter plots showing the transcription level of USF2 target genes in Jurkat^S207A^ vs. Jurkat^WT^ cells (top), and SupT1- AIMP2-N36 vs. SupT1-EGFP cells (bottom). Cells were infected by single-cycle HIV-1 for 48 h and the transcription levels of TERT, PTPN6 and TGFb1 were quantified by RT-qPCR (*P < 0.05; **P < 0.01; ***P < 0.005). (**D**) Immunoblot showing the results of co-IP assay probing the interaction between LysRS and USF2 in the nucleus in the absence and presence or HIV-1 infection. Jurkat^WT^ and Jurkat^S207A^ cells were infected by single-cycle HIV-1 at MOI 0.1 and harvested at 48 h post infection. The levels of co-IP were quantified and normalized by loading control β-actin$.$

The role of LysRS on HIV-1 transcription was investigated using a dual-luciferase system to measure the activity of the HIV-1 5′-LTR promoter (28). In this system, the reporter plasmid encodes a HIV-1 5′-LTR promoter-driven firefly luciferase gene (pGL3-LTR), and the control plasmid encodes the herpes simplex virus (HSV) thymidine kinase promoter-driven renilla luciferase gene (pTK-renilla). The activity of the LTR promoter was calculated by normalizing the firefly luciferase activity by renilla luciferase activity. Endogenous LysRS was knocked-down (LysRS-KD) in HEK293T cells by inducible shRNA expression, as previously described (17). WT LysRS and S207A- or S207D-LysRS mutants were overexpressed in the LysRS-KD cells; expression of both WT and S207A-LysRS supported translation, as reflected by the increased renilla luciferase expression compared with an empty-vector control (Supplementary Figure S2). Due to a severe aminoacylation defect, S207D-LysRS did not rescue translation of the control plasmid gene in the KD cells (Supplementary Figure S2). The dual-reporter assay showed that HIV-1 LTR promoter driven luciferase expression was activated upon overexpression of WT LysRS, but not S207A-LysRS (Figure 4A).

To test the hypothesis that the AIMP2-derived peptide sequesters LysRS in the cytoplasm, we next examined the effect of AIMP2-N36 peptide expression on nuclear localization of LysRS during HIV-1 transduction in HEK293T cells. Western blot analysis of the nuclear fraction isolated 48 h post infection showed that full-length LysRS nuclear re-localization was indeed blocked by N36 peptide expression compared with the EGFP control (Figure 4B).

Phosphorylated S207-LysRS has previously been reported to regulate USF2 target gene transcription by producing Ap4A in mast cells stimulated by IgE (22). The mRNA transcripts of USF2 target genes, telomerase reverse transcriptase (TERT), protein tyrosine phosphatase non-receptor type 6 (PTPN6), and transforming growth factor beta 1 (TGFb1), were also shown to be upregulated upon direct Ap4A transfection into cells (22). To study the potential role of LysRS in regulating USF2 in HIV-1 infected cells, we examined the mRNA transcript levels of TERT, PTPN6 and TGFb1 when the function of pS207-LysRS was blocked during HIV-1 infection. The MITF target gene granzyme B (GZMB) was also tested.

SupT1 AIMP2N36- and EGFP-expressing cells, as well as S207A KI and WT cells, were infected by HIV-1 pseudo-typed virus, followed by total RNA extraction at 72 h post infection. The transcription level of USF2 target genes was quantified by RT-qPCR and normalized by endogenous 18s rRNA. In Jurkat^S207A^ cells, the transcription of USF2 target genes PTPN6 and TGFb1 was suppressed during HIV-1 infection compared with Jurkat^WT^ cells (Figure 4C, top). In the SupT1-AIMP2-N36 cell line, reduced transcription of all three USF2 target genes was observed (Figure 4C, bottom), while MITF target gene GZMB was not affected (Supplementary Figure S3). These results support the conclusion that nuclear pS207-LysRS regulates the activity of USF2, a known activator of the HIV-1 5′-LTR (25).

USF2 was previously reported to interact with nuclear LysRS upon IgE stimulation in mast cells (40). To test the interaction between USF2 and LysRS during HIV-1 infection, a co-IP assay was performed. Endogenous LysRS was IP’d using a LysRS-specific polyclonal antibody at 48 h post infection in both Jurkat^WT^ and Jurkat^S207A^ KI cells. The Western blot results show slightly increased interaction between USF2 and LysRS upon HIV-1 infection in Jurkat^WT^ cells, but not in Jurkat^S207A^ cells (Figure 4D, middle panel). A reciprocal co-IP was consistent with full-length LysRS interaction with USF2 (Figure 4D, right panel). Taken together, these results support the hypothesis that LysRS may regulate HIV-1 transcription via USF2 activation.

### Exogenous Ap4A promotes HIV-1 expression

**Figure 5.**
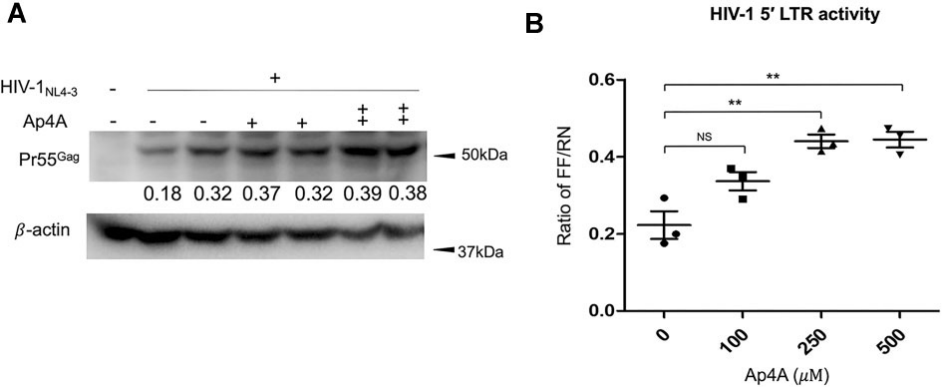
Ap4A promotes HIV-1 expression. (**A**) Jurkat cells were transfected with different amounts of Ap4A (+ 100 μM, ++ 250 μM) at 24 h post HIV-1 infection. Immunoblot shows newly synthesized pr55Gag at 72 h post infection. The pr55Gag protein levels were quantified and normalized to internal control β-actin. (**B**) Scatter plots showing the normalized HIV-1 5′ LTR activity with different concentrations of Ap4A. Jurkat cells were co-transfected with HIV-1 5′ LTR-firefly luciferase and TK-renilla constructs and different concentrations of Ap4A (100, 250, 500 μM). The dual-luciferase signals were measured at 48 h post transfection. HIV-1 5′ LTR activity is presented by the fold change of firefly luciferase over renilla luciferase signal (FF/RN) (**P < 0.01).

The regulation of HIV-1 replication by LysRS in the nucleus is likely via Ap4A synthesis, which is known to be stimulated by S207 phosphorylation (23). To directly test the effect of Ap4A on HIV-1 replication, we used liposome transfection to deliver Ap4A into Jurkat cells. Cells were infected with HIV-1_NL4-3_ and treated with different concentrations of Ap4A (100–500 μM) 24 h post infection. HIV-1 replication was promoted by Ap4A, as monitored by Gag production 72 h post infection (Figure 5A). We also used the dual-luciferase assay described earlier to quantify the transcriptional activation of the HIV-1 5′-LTR with Ap4A transfection in Jurkat cells. As shown in Figure 5B, HIV-1 5′-LTR activity was promoted with increasing levels of Ap4A, where maximal activation was observed with 250 $\mu {\mathrm{M}}$ Ap4A, consistent with the results shown in Figure 5A. We do not observe additional Ap4A stimulation of HIV-1 transcription in the presence of Tat (Supplementary Figure S4), suggesting that the primary impact of LysRS occurs early in transcription, prior to the switch to productive elongation.

### Packaging of tRNA^Lys3^ and gRNA into HIV-1 particles is facilitated by pS207-LysRS

**Figure 6.**
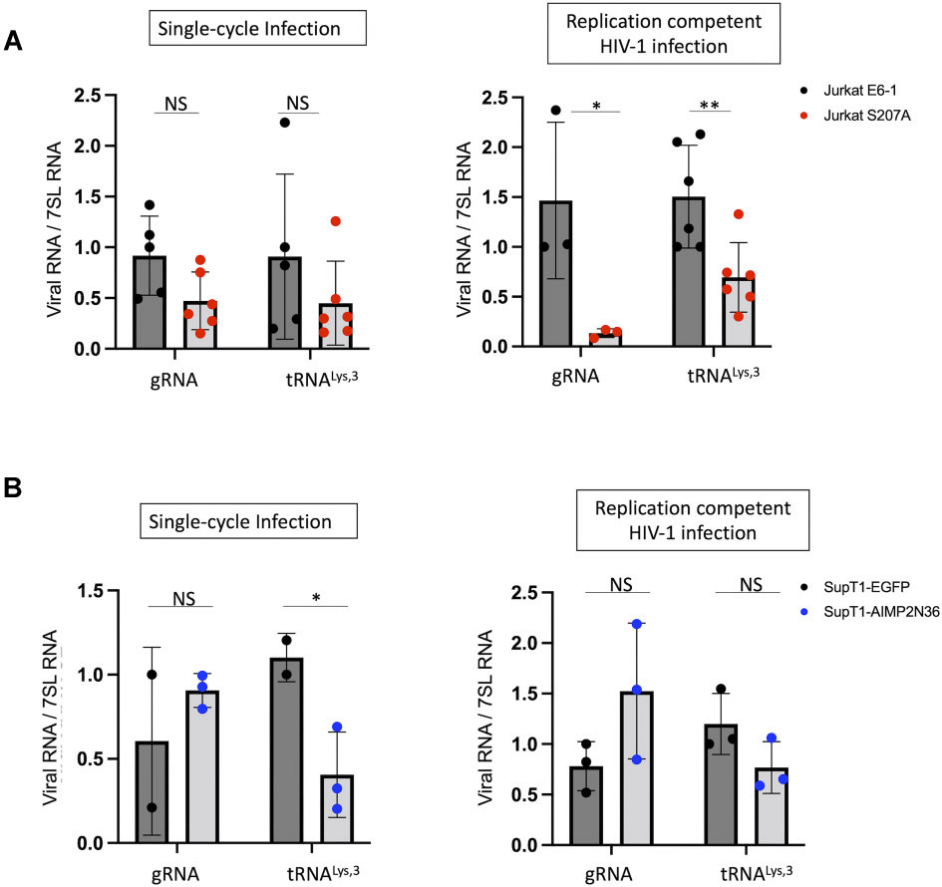
Effective packaging of tRNA^Lys,3^ and gRNA into HIV-1 particles relies on pS207-LysRS. RT-qPCR analysis of tRNA^Lys,3^ and gRNA packaging into progeny HIV-1 virions collected from Jurkat^S207A^ and Jurkat^WT^ cells (**A**) or from SupT1-AIMP3N36 and SupT1- EGFP cells (**B**). Experiments were performed with single-cycle HIV-1 infection (left) or replication competent HIV-1 infection (right) (*P < 0.05; **P < 0.01).

Using the tools developed in this study, we next examined tRNA and gRNA packaging in Jurkat^S207A^ KI and Jukrat^WT^ cells, as well as SupT1 AIMP2N36-expressing and SupT1-EGFP control cells. Cellular 7SL RNA is also packaged into HIV-1 and was used as an internal control (41,42). We observed a similar trend in the reduction of both tRNA^Lys3^ and gRNA packaging in S207A-LysRS KI cells compared to WT Jurkat cells in both single-cycle and replication-competent HIV-1 infection (Figure 6A). The decrease was statistically significant in the latter case (right graph). In SupT1-AIMP2N36 cells, the packaging of tRNA^Lys3^ was generally decreased relative to the control, with a statistically significant change observed in the single-cycle infection assays (Figure 6B). In these cells, no significant difference was observed in gRNA packaging.

## Discussion

As an HIV-1 host cell dependency factor, human LysRS has several documented functions in HIV-1 replication including facilitating primer tRNA^Lys^ packaging and HIV-1 reverse transcriptase maturation (1,12,35). Our recent study revealed the surprising finding that LysRS is partially relocalized to the nucleus upon HIV-1 infection (17). Nuclear localization depended on specific phosphorylation of S207; however, the nuclear function of LysRS in HIV-1 replication remained unclear. Here, we explored this open question and showed that pS207-LysRS regulates HIV-1 proviral DNA transcription via activation of transcription factor USF2.

A previously validated pS207-LysRS-specific polyclonal antibody (32) was used to probe endogenous LysRS phosphorylation during HIV-1 infection. These studies revealed that the major phosphorylated species was smaller than full-length LysRS (68 kDa) at ∼62 kDa; this is also the dominant species that is selectively incorporated into HIV-1 virions (16). Although the pathway of processing full-length LysRS into the ∼62 kDa truncated species is still unknown, we hypothesize that it is likely a C-terminal truncation. The N-terminus of LysRS encodes a basic domain containing the putative nuclear localization signal and is critical for high-affinity tRNA binding (15,17,43). Thus, it is reasonable that HIV-1 function does not involve a LysRS species missing the N-terminal region.

Phosphorylation on S207-LysRS is known to trigger a closed-to-open conformational change, which leads to the loss of aminoacylation function (23). *In vitro* enzymatic assays showed that S207D-LysRS could still bind tRNA^Lys^, but was not able to aminoacylate it, while the phosphoablative S207A variant maintained full aminoacylation function (17); this provided the opportunity to generate a viable S207A knock-in cell line expressing only the phosphoablative LysRS variant. Indeed, Jurkat^S207A^ KI cells generated via CRISPR/Cas9 were viable but both Gag expression and gRNA production were decreased to ∼25% of WT levels during single-cycle HIV-1 infection. A much more significant inhibition of HIV-1 replication was observed in Jurkat^S207A^ cells during a long-term spreading infection assay. The replication curve in Jurkat^S207A^ KI cells suggested that while the first round of infection was successful, progeny virions did not spread effectively. This is likely a combined effect of losing multiple functions of pS207-LysRS - HIV-1 transcriptional regulation and primer packaging.

Previous studies showed that IgE stimulation of mast cells triggered nuclear localization of pS207-LysRS, which was shown to regulate gene transcription by Ap4A synthesis and activation of transcription factors MITF and USF2 (21,22,24,44). Our results are consistent with pS207-LysRS regulation of HIV-1 transcription via activation of USF2. This transcription factor is part of a complex that binds Ras-responsive factor binding elements RBEI and RBEIII in the proviral 5′-LTR (25,45). Following T-cell receptor stimulation and Ras-MAPK signaling, USF2, together with USF1 and TFII-1, form the Ras-responsive binding factor 2 complex (RBF-2), which is recruited to the highly conserved *cis*-acting RBEIII element in the HIV-1 5′-LTR. In addition to its proposed role in stimulating transcription via chromatin remodeling, RBF-2 has also been proposed to play a role in the establishment of HIV-1 latency through a repressive function in unstimulated cells and represents a potentially important target for therapies to eliminate the latent viral reservoir (46). Interestingly, the MEK inhibitor U0126, which blocks RBF-2-dependent induction of the HIV-1 5′-LTR (25), also blocks LysRS phosphorylation and nuclear relocalization (17,25,27). Additional studies are required to investigate the potential link between LysRS nuclear function during HIV-1 infection and RBF-2 recruitment to the LTR.

Ap4A, the dinucleotide signaling molecule whose synthesis is stimulated by S207 phosphorylation of LysRS, has previously been reported to play important roles in regulating transcription and immune responses in human cells (23,24,47). Here, we showed that direct transfection of Ap4A increased HIV-1 5′ LTR activity and HIV-1 replication in the absence of Tat. Our results suggest that Ap4A might be important for early transcription or reactivation. LysRS may also impact the switch to productive transcription elongation although this possibility has not yet been tested.

Our results support the conclusion that tRNA^Lys3^ packaging into HIV-1 virions depends on pS207-LysRS. Unexpectedly, a significant decrease of HIV-1 gRNA packaging was also found in Jurkat^S207A^ cells. This inhibitory effect may be the result of the low transcription levels of HIV-1 gRNA in the host cell, resulting in the cytosolic concentration of gRNA transcripts being lower than the minimum threshold for effective packaging.

Targeting host factors that play a role in HIV-1 replication such as human LysRS is challenging if they have other essential functions in the cell. Here, we showed that a peptide, AIMP2-N36, that binds LysRS and locks it in an aminoacylation-competent conformation, can be used to suppress HIV-1 replication. This peptide only inhibits the non-canonical function of LysRS without negatively impacting its role in translation. The inhibitory effects of AIMP2-N36 on HIV-1 replication were comparable to those of the S207A-LysRS KI. In human CD4 + T cell line, expressing of AIMP2-N36 suppressed HIV-1 DNA transcription in single-cycle HIV-1 infection as well as in multiple-round replication studies.

**Figure 7.**
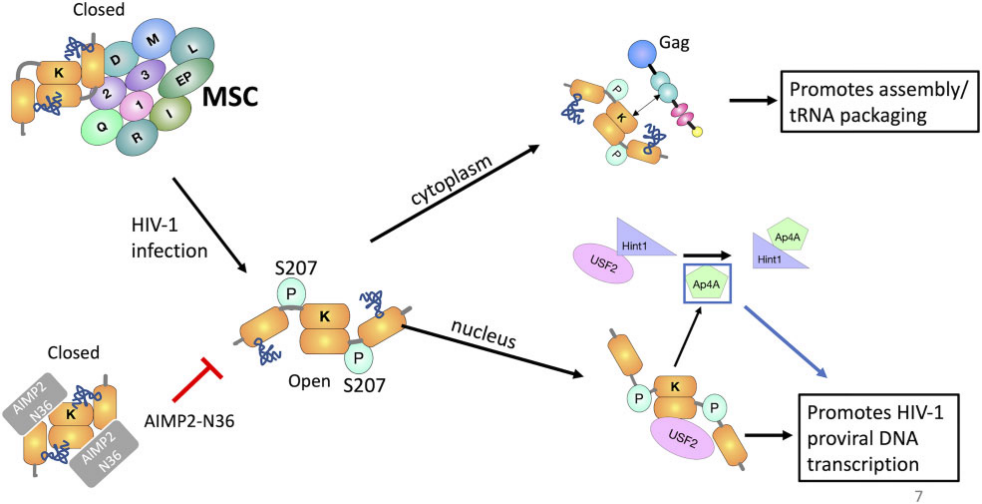
Working model for dual function of human LysRS in HIV-1 infection. Upon HIV-1 infection, LysRS is phosphorylated at S207 and undergoes a conformational change to an open conformation that is released from the MSC; this form of LysRS is able to bind tRNA but not aminoacylate it. The free cytoplasmic pS207-LysRS interacts with Gag during HIV-1 assembly to facilitate uncharged primer tRNA^Lys3^ packaging. A portion of free pS207-LysRS enters the nucleus where it promotes HIV-1 proviral DNA transcription by producing the Ap4A signaling molecule, which activates the USF2 transcription factor. The expressed AIMP2-N36 peptide binds to free LysRS, locking it in a closed conformation and blocking its non-canonical function in promoting HIV-1 replication.

The overall model for the dual function that pS207-LysRS plays to facilitate HIV-1 infection uncovered by this work is illustrated in Figure 7. Translation-competent LysRS associates with the AIMP2 scaffold protein in the MSC. HIV-1 infection triggers S207 phosphorylation and release of LysRS from the MSC and induces the closed-to-open conformational change. The open LysRS is incapable of charging tRNA but still binds tRNA and gains non-canonical functions. In the cytoplasm, pS207-LysRS interacts with Gag and facilitates primer tRNA^Lys^ packaging. In the nucleus, pS207-LysRS synthesizes Ap4A, resulting in activation of USF2 and stimulation of HIV-1 proviral DNA transcription. Overexpression of the AIMP2-N36 peptide suppresses HIV-1 replication by binding to the released pS207-LysRS and locking it in the closed conformation, preventing Gag interaction and nuclear localization. The findings presented here provide potential new anti-HIV strategies aimed at targeting host dependency factors.

## Supplementary Material

gkad941_Supplemental_File

## Data Availability

The data underlying this article are available in the article and in its online supplementary material. Further data underlying this article will be shared on reasonable request to the corresponding author.
