# Supplementary material for: Human lysyl-tRNA synthetase phosphorylation promotes HIV-1 proviral DNA transcription
Source: Nucleic Acids Res. 2023 Nov 2;51(22):12111–23. doi: 10.1093/nar/gkad941 (PMC10711549; doi:10.1093/nar/gkad941)
Supplement: gkad941_Supplemental_File [file gkad941_supplemental_file.pdf]

Human lysyl-tRNA synthetase phosphorylation promotes HIV-1 proviral DNA transcription

Yingke Tang[1,2,3], Ryan T. Behrens[5], Corine St Gelais[2,3,4], Siqi Wu[6], Saravanan Vivekanandan[7], Ehud Razin[8], Pengfei Fang[6], Li Wu[2,3,4,9], Nathan Sherer[5], Karin Musier-Forsyth[1,2,3]

[1]Department of Chemistry and Biochemistry, [2]Center for Retrovirus Research, [3]Center for RNA Biology, and [4]Department of Veterinary Biosciences, Ohio State University, Columbus OH; [5]McArdle Laboratory for Cancer Research, Institute for Molecular Virology, & Carbone Cancer Center, University of Wisconsin, Madison, WI; [6]State Key Laboratory of Bioorganic and Natural Products Chemistry, Center for Excellence in Molecular Synthesis, Shanghai Institute of Organic Chemistry, Chinese Academy of Sciences, China; [7]Cellular and Molecular Mechanisms of Inflammation Program, National University of Singapore and The Hebrew University of Jerusalem (NUS–HUJ), Singapore; [8]Department of Biochemistry and Molecular Biology, Institute for Medical Research Israel-Canada, The Hebrew University of Jerusalem, Israel; [9]Department of Microbiology and Immunology, Carver College of Medicine, University of Iowa, Iowa City, IA.

Corresponding author:
Karin Musier-Forsyth, musier-forsyth.1@osu.edu

SUPPLEMENTARY INFORMATION

Table S1. Antibodies Used in this Study.

Antibody	Source
Rabbit anti-hLysRS	Gift from Lawrence Kleiman (Lady Davis Institute for Medical Research)
Rabbit anti-pS207hLysRS	Described in Reference (Boulos et al., 2017)
Rabbit anti-HIV-1 p24 (polyclonal)	Sigma-Aldrich, SAB3500946
Mouse anti-HIV-1 p24	Thermo Lot #RX2173591
Mouse anti-GAPDH	Santa Cruz Biotechnology Lot # G2713
Rabbit anti-GAPDH	Bio-Rad
Mouse anti-β actin	Protein Tech Cat #66009
Mouse anti-α tubulin	Santa Cruz Biotechnology Lot # F2812
Mouse anti-Histone H1(AE-4)	Santa Cruz Biotechnology Lot # G2712
Mouse anti-HA	BioLegend Cat# 901503
Mouse anti-Flag	Sigma-Aldrich # F1804
Anti-Rabbit IgG (H+L), HRP Conjugate	Promega Cat# W4011
Anti-Mouse IgG (H+L), HRP Conjugate	Promega Cat# W4021
Rabbit anti-USF2	Invitrogen Cat #PA5-78217

Table S2. Sequences of qPCR primer oligonucleotides.

Target	Forward primer (5′-3′)	Reverse primer (5′-3′)
HIV-1 gRNA	TGTGTGCCCGTCTGTTGTGT	GAGTCCTGCGTCGAGAGAGC
TERT	TTGCTCCAGACACTCTTCCG	ATGTCCTTCTCGTTTAAGGGGT
GZMB	ACACCCTCCTGGAAACACTC	ATATGGCAGGCTTGGTCACT
PTPN6	CCATGGTACAGCTCTTCTGC	GGGCACTCCTAGGTTCAGGT
TGFb1	GGACCTTGTAACCAGCCGAC	CCCTGATCGCCTCCCTTCAT
$tRNA^{Lys3}$	GCCCGGATAGCTCAGTCG	TGGCGCCCGAACAGG
$tRNA^{Phe}$	GCCGAAATAGCTCAGTTGGGAGA	TGGTGCCGAAACCCGG
7SL RNA	ATCGGGTGTCCGCACTAAG	CACCCCTCCTTAGGCAACCT

Table S3. Plasmids used in this Work.

Name	Description	Source
pTripZ-shRNA	Lentivector with doxycycline- inducible TurboRFP and shRNA	Dharmacon
pCDNA3-hLysRS	Codon-optimized for expression of human LysRS in *Escherichia coli*	Described in reference (Duchon et al., 2017)
pNL4-3	Full-length HIV-1 proviral DNA	NIH AIDS Reagent Program
pNL4-3 E-R+ Luc+	HIV-1 Luciferase reporter vector	Described in reference (Connor et al., 1995)
pNL4-3 ∆Env EGFP	HIV-1 EGFP reporter vector	NIH AIDS Reagent Program
pMD2.G	Encodes VSV-G glycoprotein	Described in reference (Duchon et al., 2017)
pDeltaR 8.2	Encodes Gag-Pol, Tat, Rev	Described in reference (Duchon et al., 2017)
pGL3-LTR	Dual-luc reporter plasmid HIV-1 LTR luc	Described in reference (Antonucci et al., 2018)
pRenilla-TK	Dual-luc assay control plasmid pTK-RL	Described in reference (Antonucci et al., 2018)
pMSCV-EGFP	MSCV Lentivector, EGFP control	Described in reference (Hei et al., 2019)
pMSCV-AIMP2N36-EGFP-3xFlag	MSCV lentivector with AIMP2N36-EGFP-3xFlag construct	Described in reference (Hei et al., 2019)
pMSCV-AIMP2-3xFlag	MSCV lentivector with AIMP2-EGFP-3xFlag construct	Described in reference (Hei et al., 2019)
pMoMLV-Gag	MoMLV gag expression plasmid for lentivirus packaging	Provided by Dr. Junying Yuan (Shanghai Institute of Organic Chemistry)

Table S4. Cell lines, reagents and software

REAGENT or RESOURCE	SOURCE	IDENTIFIER
**Experimental models: Cell lines**		
HEK293T	ATCC	Cat# CRL-11268, RRID: CVCL_1926
Jurkat E6-1	NIH AIDS Reagent Program	ARP-177
THP-1	ATCC	Cat# TIB-202, RRID: CVCL_0006
SupT1	ATCC	Cat# CRL-1942, RRID: CVCL_1714
GHOST X4/R5	From Dr. Li Wu (Janas and Wu, 2009)	N/A
Jurkat ^{S207A} E6-1	This paper	N/A
Jurkat^{S207A} E6-1 TripZ-LysRS^{WT}	This paper	N/A
Jurkat^{S207A} E6-1 TripZ-LysRS^{S207A}	This paper	N/A
Jurkat^{S207A} E6-1 TripZ-LysRS^{S207D}	This paper	N/A
**Chemical reagents, culture media**		
PowerUp^{TM} SYBR^{TM} Green Master Mix	Thermo Fisher Scientific Applied Biosystems	Cat# A25742
SuperScript IV	Invitrogen	Cat# 18090050
TURBO DNase	Invitrogen	Cat# AM2239
Dual-Luciferase Reporter Assay System	Promega	Cat# E1910
TRIzol Reagent	Invitrogen	Cat# 15596018
polyethylenimine (PEI)	Thermo Fisher Scientific	Cat# BMS1003
Protease Inhibitor Cocktail	Sigma-Aldrich	Cat# P8340
phosphatase inhibitor cocktail	Cell Signaling Technology	Cat##58715
4X SDS Sample Loading Buffer	Sigma-Aldrich	
polyvinylidene difluoride (PVDF) membranes	Bio-Rad	Cat#1620177
Super-Signal chemiluminescence substrates	Thermo Fisher	Cat#34579
Lipofectamine 2000	Invitrogen	Cat# 11668019
Lipofectamine 3000	Invitrogen	Cat #L3000001
p24 ELISA kit	ZeptoMetrix	Cat#0801111
RPMI 1640	Corning	Cat# 10-040-CV
DMEM	Sigma-Aldrich	Cat# D5796
Alt-R^{TM} recombinant S.p. Cas9 nuclease-3NLS	IDT	Cat#1074181
Alt-R^{TM} CRISPR-Cas9 crRNAs	IDT	N/A
ATTO^{TM}-550 labeled Alt-R^{TM} tracrRNA	IDT	Cat#1075928
Neon® Transfection kit	Invitrogen	Cat#MPK1025
GoTaq Flexi PCR kits	Promega	Cat#M8291
NEBuilder® HiFi DNA assembly kit	New England BioLabs	Cat#E5520S
**Software and Algorithms**		
GraphPad Prism	GraphPad Software	https://www.graphpad.com/scientific-software/prism/
ImageJ	Schneider et al., 2012	https://imagej.nih.gov/ij/

FlowJo	FlowJo, LLC	https://www.flowjo.com/
QuantStudio Design and Analysis Software	Thermo Fisher Scientific	https://www.thermofisher.com/us/en/home/global/forms/life-science/quantstudio-3-5-software.html
NanoDrop 2000	Thermo Fisher Scientific	Cat# ND-2000
QuantStudio 3	Thermo Fisher Scientific<br>Applied Biosystems	N/A

Supplementary Figure S1

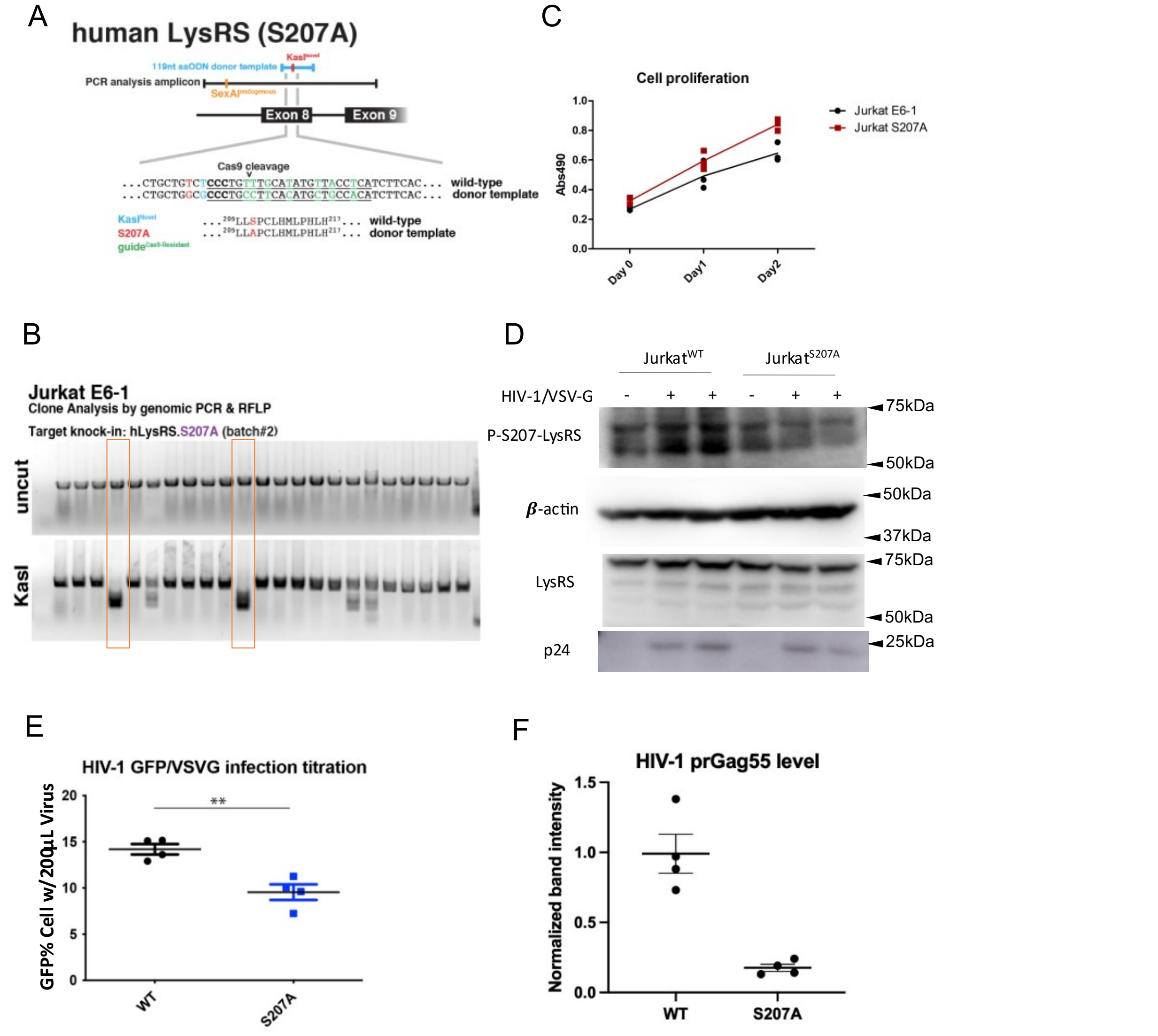

Figure S1. Generation, validation and testing of S207A knock-in Jurkat cell line. (A) The S207A knock-in Jurkat cell line (Jurkat^{S207A}) was generated using CRISPR-cas9. A homologous donor DNA segment introduced the S207A mutation and a novel KasI restriction site into both LysRS genes. (B) Homozygous knock-in was confirmed by PCR and KasI digestion. Clone 4.10D was selected for the remaining studies. (C) The viability of the JurkatS207A cell line was confirmed by an MTT assay. (D) Jurkat^{WT} and Jurkat^{S207A} cells were infected with single cycle HIV-1 virus (NL4-3 E-R+ luc/VSV-G) at MOI 5. Cell lysate was analyzed by western blotting at 72 h post infection. Phosphorylation of S207-LysRS and total LysRS were probed by using anti-pS207-LysRS antibody and anti-LysRS antibody, respectively. (E) Jurkat^{WT} and Jurkat^{S207A} cells were infected with single cycle HIV-1 GFP virus (NL4-3 dEnv-EGFP/VSV-G). HIV-1 expression levels were compared by quantifying the percentage of GFP positive cells (GFP%). (F) A quantification of immunoblotting analysis showing HIV-1 protein expression levels in JurkatS207A and JurkatWT cells during HIV-1 infection (MOI 1) at 72 hours post infection. The pr55Gag protein levels were quantified by band intensity and normalized to b-actin.

Supplementary Figure S2

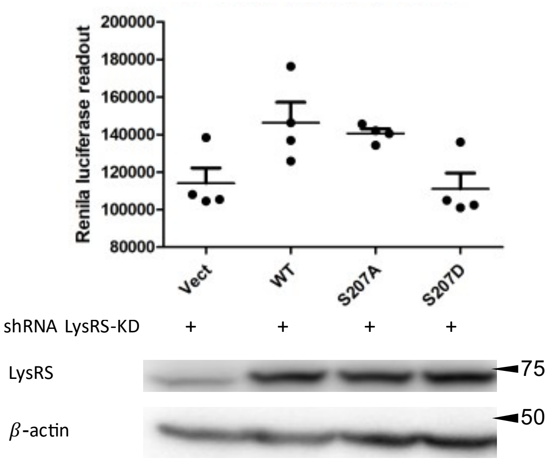

Figure S2. Translation is rescued by WT and S207A LysRS, but not S207D-LysRS. The expression of Renilla luciferase with overexpression of WT-LysRS, S207A-LysRS, S207D-LysRS or a vector control (Vect) in HEK293T cells wherein endogenous LysRS is knocked down.

Supplementary Figure S3

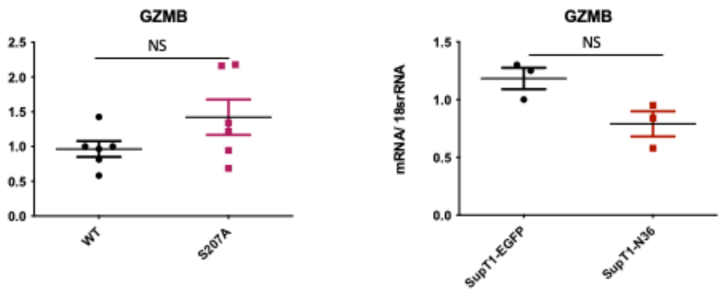

Figure S3. MITF target gene transcription. Scatter plots showing the transcription level of MITF target genes in Jurkat^{S207A} vs. Jurkat^{WT} cells (left), and SupT1- AIMP2-N36 vs. SupT1-EGFP cells (right). Cells were infected by single-cycle HIV-1 for 48 h and the transcription levels of GZMB were quantified by RT-qPCR. (NS: no significant)

Supplemental Figure S4

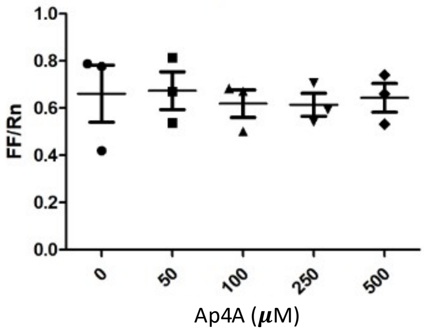

Figure S4. Ap4A has no effect on HIV-1 5′ LTR activation in the present of Tat. Scatter plot showing the normalized HIV-1 5′ LTR activity with different Ap4A concentrations in the presence of Gag and Tat. Jurkat cells were co–transfected with Ap4A and 300 ng pGL3-LTR (HIV-1 5′ LTR reporter with Firefly luciferase), 100 ng pRenilla-TK (control plasmid with Renilla luciferase) and 100 ng pCMV-dR8.2 plasmid (expressed Gag-Pol, Tat and Rev). The dual-luciferase signals were measured at 48 h post transfection.
